# Natural Products for Esophageal Cancer Therapy: From Traditional Medicine to Modern Drug Discovery

**DOI:** 10.3390/ijms232113558

**Published:** 2022-11-04

**Authors:** Jeongeun An, Soojin An, Min Choi, Ji Hoon Jung, Bonglee Kim

**Affiliations:** College of Korean Medicine, Kyung Hee University, Seoul 02447, Korea

**Keywords:** natural product, traditional medicine, herbal medicine, esophageal cancer, apoptosis, angiogenesis, metastasis, resistance

## Abstract

Esophageal cancer (EC) is one of the most malignant types of cancer worldwide and has a high incidence and mortality rate in Asian countries. When it comes to treating EC, although primary methods such as chemotherapy and surgery exist, the prognosis remains poor. The purpose of this current research is to review the range of effects that natural products have on cancer by analyzing studies conducted on EC. Fifty-seven studies were categorized into four anti-cancer mechanisms, as well as clinical trials. The studies that were scrutinized in this research were all reported within five years. The majority of the substances reviewed induced apoptosis in EC, acting on a variety of mechanisms. Taken together, this study supports the fact that natural products have the potential to act as a candidate for treating EC.

## 1. Introduction

According to the National Cancer Institute (NIH), esophageal cancer (EC) is a disease in which cancer cells proliferate in the tissue lining of the esophagus [1]. This cancer is named two different ways according to the type of malignant cell: adenocarcinoma and squamous cell carcinoma [2]. Adenocarcinoma is when a malignant cell forms in the glandular cells where fluids are released [1]. Treatments for EC include surgery, radiation therapy, chemotherapy, chemoradiation therapy, laser therapy, electrocoagulation, immunotherapy, and targeted therapy. Among these treatments, targeted therapy is still in trials and in the making [1] According to Globocan 2020, the number of new cancer cases worldwide was estimated to be 19.3 million, of which 10 million deaths were reported [3]. When it came to EC, there were 604,100 new cases and 544,076 deaths, taking up 3.1% and 5.5% of all newly arising cancer cases and deaths [3]. This indicates a positive relationship between EC and time, as it was 500,000 deaths in 2018 alone, and now, in 2020, it is close to 550,000 deaths. Incidences of EC cells in men outnumbered those in women across the world, following the same inclination with mortality rates [3]. In addition, the prevalence of esophageal squamous cell carcinoma (ESCC) over the past 5 years, 2020 being the standard, is statistically estimated to be the highest in Asia [4].

Chemoradiotherapy is commonly recommended as the primary treatment for squamous cell cancer and adenocarcinoma [5]. The standard treatment for metastatic disease for the last 40 years has been chemotherapy with cisplatin and fluoropyrimidine [6]. Recent advances in science have made available treatment options for EC approved by the FDA, including human immunoglobulins, monoclonal antibodies, humanized antibodies, and human endostatin [6]. These treatments and drugs target specific pathways or proteins actively involved in EC. According to the NIH, approximately 24% of gastroesophageal adenocarcinomas overexpress HER2, a growth factor receptor [7]. The epidermal growth factor receptor (EGFR) pathway is also crucial in cancers for it encourages metastasis and tumor growth [8]. Vascular endothelial growth factors (VEGFs) play an important role in regulating the proliferation and angiogenesis of vascular endothelial cells [9]. Hepatocyte growth factor receptor (c-Met) is a receptor for hepatocyte growth factor (HGF), which regulates tumor cell growth, invasion, metastasis, and angiogenesis [10]. Although there are numerous drugs available to combat EC, there are various forms of side effects. Examples of the side effects include vomiting, nausea, fever, perforations, severe infection from within, diarrhea, constipation, and allergic reactions [11]. The aforementioned FDA-approved drugs paired with chemotherapy seem to result in higher efficacies in diminishing the EC tumor burden in patients [12]. Trastuzumab is a manmade version of an immune system protein that targets HER2 that is used to treat EC [6]. However, trastuzumab causes low blood cell counts, which can increase a person’s risk of infection and bleeding [6]. Other common side effects of FDA-approved drugs include loss of appetite, fatigue, and hair loss [6].

All cancers have the tendency to metastasize, where, according to the NIH, cancer cells break away from the primary location on to secondary and tertiary locations [13]. They do so by forming new blood vessels, which is called angiogenesis [13]. In addition, therapeutics do not always work on cells that are malignant [14]. Cancer cells grow resistant towards certain drugs to prevent metastasis and angiogenesis [14]. Therefore, in order to prevent cancer cells from proliferating, apoptosis, anti-resistance, anti-angiogenesis, and anti-metastasis are important [15]. Normally, cells that find themselves failing to function according to changes in body conditions turn on self-cell death. However, most of those that do not recognize their failure are soon aggregated into malignantly functioning cells that do not go through self-cell death and form cancer [16].

Currently, diverse research and experiments are being conducted on the potential of natural products as novel cures for ESCC. Most of the research that has been released include herbal medicines often used in traditional Korean medicine (TKM) and traditional Chinese medicine (TCM) [17]. The common constituent that is often found to be effective in being anti-ESCC is flavonoid [18]. In the past decade, there have been numerous research works conducted on leading out the purpose of finding cancer-curing natural products. One of the famous findings effective in alleviating ESCC tumor burden was green tea [19]. Green tea extracts are not only a well-known panacea, but also used in traditional medicinal backgrounds as it prevents disease, promotes glucose metabolism, and alleviates inflammation [20]. Once underestimated for their curing abilities, natural extracts and products have revolutionized the drug field often dominated by science as they contain little to no synthetic compounds, optimizing particular biological functions in vivo. In addition, natural products that were recently researched are only the tip of the iceberg when it comes to their medical potential and innumerable diversity that is yet to be touched upon [21].

Thus, the goal of this study is to initiate a process to grasp the range of effects natural products have on cancer. The focus is to examine the types of research conducted on EC where natural products have been effective in bringing results of four aforementioned anti-cancer effects. This paper takes a deep dive into EC in order to identify the anti-cancerous mechanisms of natural products.

## 2. Anti-Esophageal Cancer Effect of Natural Products

### 2.1. Apoptosis and Natural Products

Natural compounds ranging from alkaloid to triterpene, extracts, and decoction—water derived from crude drugs—exhibit apoptotic properties on esophageal squamous cell carcinoma (Table 1).

Jiang et al. showed the apoptotic effects of matrine (MT) on esophageal squamous cell carcinoma [22]. MT exhibited maximum effect on increasing reactive oxygen species (ROS) activity to make an apoptotic-favorable environment, along with mitochondrial failure. In a dose-dependent fashion, mitochondrial activity of esophageal cancer cells visibly decreased, which potentially led to apoptosis. Thus, apoptosis and mitochondrial-dependent apoptosis came into effect by MT’s attenuation of Bcl-2 and accretion of Bax, cleaved caspase-3, -8 and -9 levels.

Wang et al. reported that matrine (MT) induces apoptosis and cell cycle arrest and inhibited tumor formation [23]. Cleaved caspase-3, -9, and cleaved PARP were upregulated, and Bcl-2/BID was downregulated. EC109 cells were subcutaneously injected into nude mice and MT was injected every 2 days for 3 weeks. Cell cycles of esophageal tumor cells were halted at G0/G1phase. In addition, MT located itself in the nucleus of the tumor cells, causing apoptosis and inhibiting tumor formation.

An et al. showed the apoptotic effects of neferine (Nef) on esophageal squamous cancer cells [24]. The apoptotic behavior was evident through the ROS-mediated JNK signal route. Levels of cleaved caspase-3, -9, and cleaved PARP were increased with the use of Nef, resulting in the cleavage of cancer cells, and ultimately leading to programmed cell death. Along with the apoptotic effects, treatments of Nef aided in inhibiting cell proliferation and cell cycle arrest.

Zhu et al. claimed that osthole (OST) aids in targeting EC cancer cells in various ways, but most importantly in cell cycle arrest and apoptosis [25]. OST induced the reduction in proliferation through the concentration control of PTEN and PI3K/AKT in KYSE150 and KYSE410 cells. EC density vastly decreased after OST treatment. Cell cycle arrest happened at the G2/M phase by the upregulation of Bax and downregulation of Bcl-2 proteins.

Yang et al. detailed TR35, a fraction from Xinjiang Bactrian camel milk, as containing anti-cancer properties on esophageal cancer cells [26]. TR35 on EC109 showed significant decrease in cell viability and nuclear fragmentation, indicating apoptotic performances. Cleaved caspase-3 activity increased, strengthening its pro-apoptotic effects on esophageal squamous cancer cells. In vivo, mice treated with TR35 displayed visible esophageal tumor size and weight reduction due to the attenuation of HSP60.

Almanaa et al. claimed curcumin (CU) as a plausible novel remedy for esophageal cancer [27]. CU exerts eliminating properties of cancer stem cells. When exposed to CU, YES-2 cells turned out to have the least survival rate compared to other 5 human esophageal squamous cancer cell lines. The levels of stem cell markers such as ALDH1A1 and CD44 seemed to differ between each of the esophageal cell lines, but increased levels of CU aided the riddance of cancer stem cells.

Chen et al. charted the anti-proliferative effects moscatilin has on esophageal cancer cells in vivo [27]. Moscatilin intraperitoneally injected into mice prompted significant tumor size reduction, indicated by the activity of protein phospho-Plk1. Numerous cells showed signs of apoptosis with dense nucleus chromatin fragments and condensed cytoplasm. Radiation partnered with preincubation of esophageal cancer cells with Moscatilin caused a reduction in colony formation compared to without.

Wang et al. argued that acetyl-macrocalin B (A-macB) demonstrates suppression of cancer growth in esophageal squamous cell carcinoma [28]. Non-cancer-favorable intracellular ROS activity increased with a dose of 20 μM of A-macB for 24 h. A-macB and AZD7762 were seen blocking cell cycle implementation at G2/M stage in vivo. 9 days after tumor cell transplantation, 12 mg/kg of A-macB was treated for 29 days.

Shi et al. reported the pro-apoptotic effects of JDA-202, a natural compound isolated from Isodon rubescens, on esophageal cancer cells [29]. A dose of 20 μM of JDA-202 on EC cells alone for 24 h induced apoptosis. EC109 cells injected into subjective mice were treated with 20 mg/kg of JDA-202 for 21 days. JDA-202, in effect, attenuated the expression of Prx I, the recipe to the development of carcinoma cells. Moreover, cell cycle arrest was encouraged and assisted by JDA-202.

Song et al. claimed that oridonin (ORI) inhibits esophageal squamous cell carcinoma growth [30]. This effect was found to be effective through down-regulation of cyclin B1, Bcl-2, and AKT both in vivo and in vitro. When these KYSE70 cells were injected into the mice, 40 and 160 mg/kg of ORI for 52 days showed visible inhibition of cancer cell growth. Specifically, EC cell-specific-protein AKT1 and AKT2 expression were halted.

Ma et al. claimed the strong apoptotic potential of jaridonin on esophageal squamous cell carcinoma [31]. EC cells exposed to jaridonin experienced cell cycle arrest during the G1/S phase accompanied by cell shrinkage and condensation, which refers to the initiation of apoptosis. Major tumor suppressor genes Bax and p53 were also activated and in turn, cytochrome c concentration, cleaved caspase-3, and cleaved caspase-9 were detected.

Wang et al. reported that flavonoid Baohuoside-I (Icariin-II) works in inhibiting cell growth when it comes to esophageal squamous cancer cells [32]. In vitro, Icariin-II exhibited effective reduction in cell proliferation due to the decrease in β-catenin gene expression. Cyclin D1 and survivin were downstreamed with the use of Icariin-II. In addition to proliferating effects of Icariin-II, apoptosis was induced in a dose dependent manner.

Fan et al. reported that icariin (ICA) has anti-esophageal-cancer properties that inhibit cell proliferation and induces apoptosis [33]. ICA on KYSE70 showed attenuating effects on cancer colony formation and cell viability. A clear reduction in esophageal tumor sizes was visible due to cell cycle arrest at G2/M stage and an increase in Matrix metallopeptidase (MMP) resulted in increased levels of ROS in vivo. PI3K/AKT signal was halted, downregulating ZO1, N-cadherin, ZEB1, Slug 1, phospho-PI3 Kinase p85, phospho-AKT, signal transducer and activator of transcription3 (STAT3), and Ki-67, which moderated metastasis and invasion.

Ye et al. reported the anti-metastatic effects of isoliquiritigenin (ISL) as a novel remedy to treat esophageal cancer [34]. For 72 h, 20 μM of ISL resulted in the lowest survival number of cancer cell colonies in various esophageal squamous cancer cell lines by downregulating AP-1 and cyclin D1. In vivo, mice were intraperitoneal injected with ISL. This resulted in the attenuation of phosphorylation of EGFR and intensity of Ki-67, both which are markers of proliferation in cancer cells.

Guo et al. claimed that proanthocyanidin extract from grape seed (GSPE) inhibits esophageal squamous cancer cells from proliferating by inducing apoptosis specifically through the control of the nuclear factor kappa B (NF-_K_B) signal pathway [35]. Exposure to GSPE led to a higher percentage of EC109 cells entering the early apoptosis stage through the activation of the NF-_K_B signaling pathway. In addition, the migration radius of EC cells significantly shortened, followed by the attenuation of IL-6 and COX-2, along with the inversely proportional concentration of Bcl-2 and Bax.

Zheng et al. reported the apoptotic effects of quercetin (QC) [36]. Through immunofluorescence examinations, esophageal cancer cells exposed to QC showed signs of apoptotic behavior. When esophageal cancer cell lines were exposed to QC along with CD133, pro-apoptotic behavior was visible, specifically in the cytomembranes. The main signaling cascade to self-induced cell death, NF-_K_B and histone deacetylases 1 (HDAC1), was downregulated, exhibiting properties of apoptosis on a nuclear level.

Gao et al. reported the use of genistein (GNT) as an effective inhibitor of proliferation and promotor of apoptosis in esophageal squamous cancer cells [37]. GNT caused a change in the densities of cell apoptosis-related genes and the arrested cell cycle at G0/G1 phase. Apoptosis-inducing Bax increased while anti-apoptosis protein Bcl-2 decreased. In mice, cell cycle-related genes CDK4/6 and cyclin D1 were down-regulated. A long-term treatment of GNT vastly reduced EGFR expression, which cut off the AKT and Janus kinase 1/2 (JAK1/2) signaling for proliferation.

Huang et al. reported that pristimerin (PM) induces apoptosis, cell cycle arrest, and autophagy in esophageal cancer cells [38]. In the presence of PM, the activity of cleaved caspase-3, -9, Bax, and CDKN1B increased, while that of Bcl-2, cyclin E, CDK2, and 4 decreased. In in vivo experiments, subcutaneously injected PM arrested the cell cycle at the G0/G1 phase by the alteration of key molecules. PM especially acted upon CDKN1B, upregulating it and, thus, contributing to the nonfulfillment of cell proliferation.

Wu et al. reported the synergistic potentials of terrein (TR) with cisplatin, along with its antitumor effects, emphasizing its proliferating ability [39]. EC109 cells exposed to TR resulted in a reduction in cancer cell proliferation along with cell cycle arrest at the G2/M phase. This was possible through the downregulation of cyclin B1 and CDC2, which allowed the arrest of keratinocytes. The amount of 5 µM of cisplatin exhibited the highest enhanced anti-proliferative effects with TR.

Lu et al. demonstrated that isoalantolactone (IAL) induces apoptosis in esophageal tumor cells [40]. Treatment of IAL increased the expression of cleaved caspase-7, cleaved PARP, DR5, and ROS, and decreased the expression of procaspase-10. However, IAL is selectively cytotoxic to varying types of esophageal cancer cells and, thus, is more effective towards EC109 than L-O2 cells. IAL subcutaneously injected into the mice led to the overproduction of ROS and upregulation of DR5, initiating apoptosis.

Zhang et al. reported the anti-metastasis and apoptotic effects of germacrone (GM) [41]. A dose of 30 μg/mL of GM for 24 h showed visible effects of apoptosis on EC109 and EC9706. Specifically, GM prompted intrinsic apoptosis through the downregulation of Bcl-2 levels. Expressions of Bax, and cleaved caspase-3, -9, -12, and -7 are closely affiliated to microenvironmental stress within a cell, which contributes to apoptotic behavior in esophageal cancer cells.

Meng et al. presented a combination therapy of natural borneol (NB) and paclitaxel (PTX) for treating EC cells as they enhance PTX-induced apoptosis through their synergistic effects [42]. PTX alone exhibited its anti-metastatic effect in 6 h after exposure. With the addition of NB, within 3 h, esophageal squamous cancer cells lost their viability. A longer exposure to PTX firsthand, paired with a larger dose of NB, showed significant anti-cancer effects through the activation of cleaved caspase-3 and downregulation of p-AKT.

Ma et al. reported the use of gypenoside L (GP-L) as an anti-metastasis remedy for esophageal squamous cancer cell [43]. GP-L resulted in senescence of esophageal cancer cells. EC109 cell cycle was arrested at the S phase through the inhibition of proteins p21, p27, and p18. MAPK and NF-_K_B signaling pathways, autophagy, and ROS were shown to coherently contribute to anti-proliferative potential of GP-L.

Yan et al. claimed that Rhizoma Paridis Saponins (RPS) suppresses tumor growth in the esophagus of rats [44]. RPS reduced expression of cyclin D1 in rats previously injected with EC9706 cells. Two different doses of RPS were injected into the rats for 24 weeks. Not only did RPS inhibit the viability, migration, and invasion of esophageal cancer cells, but it also induced apoptosis and cell cycle arrest. Specifically, COX-2 expression was reduced through the release of PGE2 by RPS.

Vahedi et al. reported avocado (AV) as an anti-cancer extract for esophageal squamous cell carcinoma [45]. A dose of 20 μg/mL of AV extract for 48 h led to the lowest percentage of viable cells. In this study, a comparison of the effects of AV extract on esophageal squamous cancer cells and colon adenocarcinoma cells was conducted. Among the types of extracts from AV, ethyl acetate extract was statistically proven to have the lowest cell viability, showing the most anti-cancer effect.

Wang et al. demonstrated that black raspberry (BRB) has anti-inflammatory, apoptotic, and anti-angiogenetic effects in esophageal carcinogenesis [46]. A diet containing 5% BRB powder was given to F344 rats. BRB influenced the COX pathway of the arachidonic acid metabolism by downregulating COX-2 and PGF2 levels. In addition, BRBs were found to affect the LOX pathways of arachidonic acid metabolism through the expression of MMP10, inhibiting LTB4. The study also reported that the expression of genes can be influenced by BRB during the late stages of rat esophageal cancer.

Kresty et al. reported cranberry (CB) as a potential anti-cancer remedy for esophageal squamous cancer cells [47]. In OE19 esophageal squamous cancer cells, CB significantly induced necrosis by 40%. In JHAD1 cells, cell death was significantly induced. For both cells, autophagy could be seen where ultrastructural changes were caused on the esophageal squamous cancer cells. With the oral treatment of CBs, OE19 cells in mice reduced in size and weight and turned on the AKT/mTOR/MAPK signaling pathway, aiding cancer cell apoptosis.

Zhao et al. reported Fructus forsythia (FF) as a novel extract that induces apoptosis in esophageal cancer cells [48]. The use of FF resulted in the attenuation of the anti-apoptotic Bcl-2 family. In addition, an intrinsic apoptotic pathway was activated through the mitochondria by FF. There was a significant decrease in the tumor size and weight of the FF treated mice compared to the control one.

Li et al. demonstrated the restricting effects of green husk on esophageal squamous cancer cell lines [49]. Green husk extractives caused apoptosis, reduction in proliferation, and migration. The EtOH extract, EtOAc soluble fraction, and gallic acid (GA) extractives impaired the motility and invasion of cancer cells by prohibiting migration and proliferation by the downregulation of MMP2, MMP9, Bax, cleaved caspase-3, and Bcl-2. Cell cycle arrest was initiated through the attenuation of cyclin D1.

Fan et al. claimed the anti-angiogenetic effects of Marsdenia tenacissima extract (MTE) [50]. KYSE150 and EC109 exposed to MTE exhibited vast attenuation in the migration span. MTE inhibited esophageal squamous cancer cell proliferation by arresting the cell cycle at the G0/G1 phase through the decrease in cell cycle proteins cyclin D1/D2/D3, CDK4/6, and cyclin-dependent kinase inhibitors (CDKIs) p18, p21, and p27. In addition, AKT and MAPK signals were blocked.

Li et al. reported the apoptotic-inducing and anti-proliferating property of Paris Polyphylla Smith extract (PPSE) [51]. The exposure of esophageal squamous cancer cell to PPSE yielded high concentrations of connexin 26 expression, leading to an anti-cancer environment. This was found to be effective in being pro-apoptotic as Bad is increased, when connexin 26 has already weakened the cancer cell population.

Peiffer et al. reported that black raspberry (BRB) has anti-inflammatory and tumorigenesis effect in esophageal cancer cells [52]. An experimental diet containing 6.1% of BRB, black berry polyphenolic anthocyanin, and PCA, a major metabolite of black berry polyphenolic anthocyanin, was given to F344 rats. This study confirmed the inhibitory effects of BRB seeing from the deactivation of COX-2, and activation of NF-_K_B, PTX3, and sEH. The results demonstrated that the whole BRB was most effective in reducing esophageal tumorigenesis.

Hadisaputri et al. reported that rhizome extract from *Curcuma zedoaria* (CZ) has apoptotic effect on esophageal carcinoma cells [53]. TE-8 and HET-1A cells treated with CZ extract activated cleaved caspase-9, cleaved caspase-3, PARP, and PTEN and deactivated Bcl-2, MMP-2, AKT, FGFR1, and STAT3. It was proved that CZ extract subcutaneously injected into BALB/c mice contains suitable constituents that modulate multi-targets against apoptotic proteins in esophageal tumor cells.

Skerman et al. reported the use of Sutherlandia spp. extract, Sutherlandia frutescens, and Sutherlandia tomentosa as a novel anti-cancer remedies for esophageal squamous cancer cells [54]. Cell viability significantly reduced following exposure to Sutherlandia spp. extracts. SNO cancer cells were misstructured, including swelling and blebbing of plasma membrane, signifying apoptosis. The concentration of cleaved caspase-3 and -7 increased, which demonstrates the induction of caspase-dependent cell death.

Nagata et al. reported the anti-cancer effects of daikenchuto (DKT) [55]. Esophageal cancer cell lines KYSE520 and KYSE790 exposed to DKT decreased cancer cell viability in a time-dependent manner, with 72 h being the most favorable. A total of 20 µg/mL of DKT peritoneally injected in mice halted cancer cell proliferation by enhancing the PAK1/cyclin D1 pathway in neurofibromatosis-derived tumor cells and blocking the signal mainly enacted by protein AKT/AR.

Liu et al. reported the apoptotic effects of Rosa roxburghii Tratt (CL) and Fagopyrum cymosum (FR), both separately and in combination with esophageal squamous cell carcinoma [56]. CL and FR exhibited varying results, where pro-apoptotic effects were distinguishably higher when cells were exposed to CL compared to FR. However, when CL and FR was exposed to CaEs-17 cells in combination, apoptotic effect almost doubled compared to when the two compounds were separately experimented. The effect was seen through the upregulation of Bax and down regulation of Bcl-2 and Ki-67.

Jia et al. reported the anti-metastatic effects of tonglian decoction on EC109 cells [57]. EC109 cells exposed to tonglian decoction exhibited inhibitory effects on cell proliferation. Cells were arrested at the G1 phase and drawn back from entering the S and G2/M phase. In addition, the expressions of significant proteins that are a part of the NF-_K_B signal pathway, which is only activated in esophageal squamous cancer cells, such as TNF-α and IL-1β, were inhibited.

**Figure 1:**
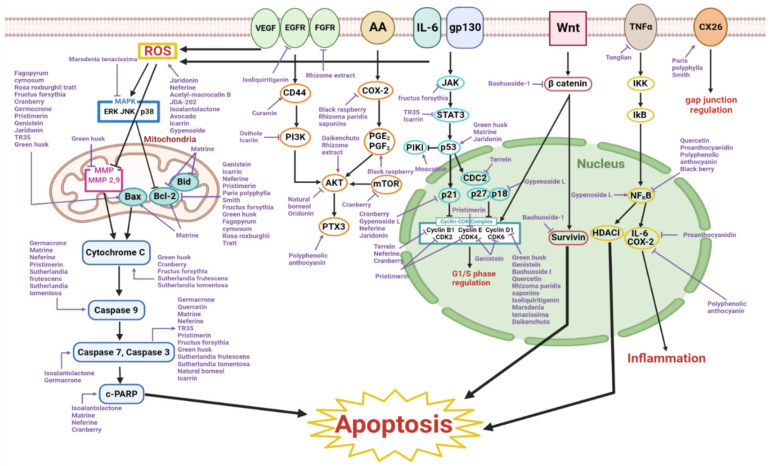
Schematic diagram of apoptosis-inducing natural products in Esophageal Cancer.

Apoptosis, also known as programmed cell death, acts as a hallmark of cancer and inhibits tumor development. Thirty-seven substances that were researched were reported to induce apoptosis in esophageal cancer. The majority of the natural products that had apoptotic properties on esophageal cancer were organic compounds found in plants. Extracts, fruits, and decoctions were also present. The esophageal cancer cell lines that were frequently used for studies the regarding apoptotic effects in vitro were KYSE30, KYSE-70, KYSE-150, KYSE-450, HET1A, EC109, EC9706, and TE1. BALB/c mice were mainly used as animal models for research in vivo. Among apoptosis-inducing natural products, matrine, neferine, osthole, TR35, curcumin, acetyl-macrocalin B, JDA-202, oridonin, jaridonin, baohuoside-I, icariin, isoliquiritigenin, proanthocyanidin, quercetin, genistein, pristimerin, terrein, isoalantolactone, germacrone, natural borneol, gypenoside L, *Rhizoma paridis saponins*, avocado, black raspberry, *Fructus forsythia*, green husk, *Marsdenia tenacissima*, rhizome extract, *Sutherlandia frutescens*, *Sutherlandia tomentosa*, Daikenchuto, *Rosa roxburghii Tratt*, *Fagopyrum cymosum*, and Tonglian had little to no cytotoxicity in normal cells [22,23,24,25,26,27,28,29,30,31,32,33,34,35,36,37,38,39,40,41,42,43,44,45,46,48,49,50,53,54,55,56,57]. The rest of the research did not include cytotoxicity tests. Matrine, neferine, osthole, TR35, curcumin, jaridonin, isoliquiritigenin, proanthocyanidin, quercetin, germacrone, natural borneol, gypenoside L, avocado, cranberry, green husk, *Marsdenia tenacissima*, *Paris polyphylla Smith*, Sutherlandia frutescens, *Sutherlandia tomentosa*, *Rosa roxburghii Tratt*, *Fagopyrum cymosum*, and Tonglian were only researched in in vitro experiments, thus illustrating the need for the investigation of anti-cancer effects in mice models for these substances [22,24,25,26,27,31,35,36,39,41,42,43,45,47,49,50,51,54,56,57]. Research regarding moscatilin, black raspberry, and polyphenolic anthocyanin were only researched in in vivo experiments, hence requiring in vitro experiments for further investigation [46,52,58]. Besides their apoptotic effects on esophageal cancer, several substances had inhibitory effects on tumor formation, cell proliferation, inflammation, and cancer stem cells. Some of the substances induced autophagy and also cell cycle arrest. Few of the natural products were effective in preventing angiogenesis and metastasis. The anti-cancer mechanisms of the natural products are elucidated in Figure 1.

**Table 1:** Apoptosis-inducing natural products.

Classification 1	Classification 2	Compound/Extract	Source		Experimental Model	Dose; Duration	Efficacy	Mechanism	Reference
Single Compound	Alkaloid	Matrine	*Sophora flaavescens*		KYSE150	1, 2, 3 mg/mL; 24 h	Induction of apoptosis	↑ ROS, Bax, c-caspase-3, c-caspase-8, c-caspase-9 ↓ Bcl-2	[22]
Single Compound	Alkaloid	Matrine	*Sophora flaavescens*		EC109	0.25, 0.5, 0.75, 1, 1.5, 2 mg/mL; 24 h	Induction of apoptosis inhibition of tumor formation	↑ c-caspase-3, c-caspase-9, c-PARP ↓ Bcl-2/BID	[23]
					BALB/c mice	1.25, 2.5, 5, 10 mg/kg; 26 d			
Single Compound	Alkaloid	Neferine	*Nelumbo nucifera* (lotus)		KYSE30, KYSE150, KYSE510	10, 15, 20 μM; 24 h	Induction of apoptosis	↑ ROS, cyclin B1, c-caspase-3, c-caspase-9, PARP ↓ Nrf2, p21, Bcl-2	[24]
					BALB/c mice	10 mg/kg; 24 d			
Single Compound	Coumarin	Osthole	*Cnidium monnieri* (L.) Cusson		KYSE30, KYSE150, KYSE180, KYSE410, KYSE450	80, 120, 160 μM; 48 h	Induction of cell cycle arrest and apoptosis	↑ PTEN ↓ PI3K/AKT	[25]
Single Compound	Dairy	TR35	Xinjiang Bactrian camel milk		EC109	1, 2, 4 mg/mL; 48 h	Induction of apoptosis	↑ c-caspase-3, Bax ↓ HSP60, HSPA8, HSP27, DJ-1, PRDX1, NOB1, CAP1, GAPDH, Actin, PHGDH, MAPRE1, PDXK	[26]
					BALB/c mice	4 mg/mL; 55 d			
Single Compound	Diarylheptanoid	Curcumin	*Curcumin* *longa*		TE1, TE8, KY5, KY10, YES1, YES2	20, 40, 60, 80 μM; 30 h	Elimination of cancer stem cells	↑ ALDH1A1, CD44	[27]
Single Compound	Dendrophenol	Moscatilin	*Dendrobium, Dendrobium loddigesii*		Nude mice	50 mg/mL; 2 m	Inhibition of cancer growth	↑ p-Plk1	[58]
Single Compound	Diterpene	Acetyl-macrocalin B	*Isodon silvaticus* (C.Y.Wu & H.W.Li) H.W.Li		KYSE30, KYSE450	10, 15, 20 μM; 24 h	Suppression of cancer growth	↑ ROS, AZD7762	[28]
					BALB/c mice	12 mg/kg; 29 d			
Single Compound	Diterpene	JDA-202	*Isodon rubescens* (Labiatae)		EC109, EC9706, KYSE450, HET1A	10, 20 μM; 24 h	Induction of apoptosis	↑ ROS ↓ Prx I	[29]
					BALB/c mice	20 mg/kg; 21 d			
Single Compound	Diterpene	Oridonin	*Rabdosia rubescens*		KYSE70, KYSE410, KYSE450	5, 10, 20 mmol/L; 72 h	Inhibition of cancer growth	↓ cyclin B1, Bcl-2, AKT	[30]
					SCID mice	40, 160 mg/kg; 52 d			
Single Compound	Diterpenoid	Jaridonin	*Isodon* *rubescens*		EC9706, EC109, EC1	10, 20, 40 μM; 24 h	Induction of apoptosis and cell cycle arrest	↑ p53, p21, Bax, cyt- c, c-caspase-9, c-caspase-3, ROS, L-NAC	[31]
Single Compound	Flavonoid	Baohuoside-I			EC109	12.5–50 μg/mL; 48 h	Inhibition of cell growth	↓ β-catenin, survivin, cyclin D1	[32]
					BALB/c mice	25 mg/kg; 3 w			
Single Compound	Flavonoid	Icariin	*Herba Epimedii*		KYSE70	20, 40, 80 μ M; 48 h	Inhibition of cell proliferation and induction of apoptosis	↑ ROS ↓ ZO1, N-cadherin, ZEB1, Slug1, p-p85, p-AKT, p-STAT3, Ki-67	[33]
					Nude mice	40 μg/g; 4 w			
Single Compound	Flavonoid	Isoliquiritigenin	Licorice root		KYSE140, KYSE520, TE1	5, 10, 20 µM; 24, 48, 72, 96 h	Inhibition of esophageal squamous cell carcinoma	↓ EGFR, ERK1/2, AKT, AP-1, cyclin- D1	[34]
Single Compound	Flavonoid	Proanthocyanidin	*Vitis vinifera* L.		EC109	25, 50, 80 μg/mL; 24 h	Induction of apoptosis and inhibition of cell proliferation	↑ Bax, c-caspase-3 ↓ IL-6, CRP, COX-2, Bcl-2, PGE2	[35]
Single Compound	Flavonoid	Quercetin			EC109, EC9706	40 μmol; 48 h	Induction of apoptosis	↑ c-caspase-3 ↓ NF-_K_B, HDAC1, cyclin D1, RELA	[36]
Single Compound	Isoflavonoid	Genistein	Fabaceae		HET1A, EC109, EC9706, CaES17	5, 15, 12, 125 μM; 48 h	Inhibition of cell proliferation and promotion of apoptosis	↑ Bax ↓ CDK4, CDK6, cyclin D1, Bcl-2, EGFR	[37]
					Nude mice	5 mg/kg, 10 mg/kg; 42 d			
Single Compound	Quinone	Pristimerin	*Celastraceous, Hippocratic*		EC9706, EC109	0.5, 1.0, 1.5, 2.0 μmol/L; 48 h	Induction of apoptosis, cell cycle arrest, and autophagy	↑ c-caspase-3, c-caspase-9, Bax, CDKN1B ↓ Bcl-2, cyclin E, CDK2, CDK4	[38]
					Nude mice	1.5 μmol/L; 48 h			
Single Compound	Polyketide	Terrein	*Aspergillus terreus*		MCF7, HeLa, EC109	2.5, 5, 10, 20, 40 µM; 3 d	Inhibition of cell proliferation	↓ cyclin B1, CDC2	[39]
Single Compound	Sesquiterpenoid	Isoalantolactone	*Inula helenium* L.		EC109, EC9706, TE1, TE13, LO2	20, 30, 40 μM; 24 h	Induction of apoptosis	↑ c-caspase-7, c-PARP, DR5, ROS ↓ procaspase-10	[40]
					BALB/c mice	40, 80 mg/kg; 24 d			
Single Compound	Terpene	Germacrone	*Saussurea costus*		EC109, EC9706, HET1A	10, 20, 30 μg/mL; 24 h	Inhibition of cell proliferation and induction apoptosis	↑ Bax, c-caspase-3, c-caspase-9, c-caspase-12, c-caspase-7 ↓ Bcl-2	[41]
Single Compound	Terpene	Natural Borneol	camphor		HET1A, TE1, TE13	20, 40, 80 μg/mL; 24, 48, 72 h	Enhancement of paclitaxel-induced apoptosis	↑ c-caspase-3 + PTX ↓ AKT, survivin	[42]
Single Compound	Triterpene	*Gypenoside* L.	*Gynostemma pentaphyllum* (Thunb.) Makino		HepG2, EC109	20, 40, 80 μM; 24 h	Inhibition of proliferation	↑ MAPK, NF-_K_B ↓ p21, p27, p18	[43]
Single Compound	Triterpene	Rhizoma Paridis Saponins (RPS)	*Rhizoma Paridis*		EC9706, KYSE150	5, 10, 20 μg/mL; 24 h	Suppression of cancer growth	↓ cyclin D1, COX-2	[44]
					F344 rats	350, 100 mg/kg; 24 w			
Mixture	Fruit extract	Avocado			KJSE30	20 μg/mL; 48 h	Inhibition of cancer cell growth	↑ ROS	[45]
Mixture	Fruit extract	Black raspberry			F344 rats	5%; 35 w	Inhibition of inflammation, apoptosis, and angiogenesis	↓ MMP10, COX-2, PGF2	[46]
Mixture	Fruit extract	Cranberry			JHAD1, OE19, OE33	50, 100 µg/mL; 24, 48 h	Inhibition of pleiotropic cell death induction	↑ Bax and cyt c, p21, cyclin B1, PARP ↓ cyclin A, PCNA, RTK, BCL-xL	[47]
					NU/NU athymic mice	250 μg; 6 d			
Mixture	Plant extract	Fructus forsythia			TE1, TE13, EC109, YES2	0.25, 0.50, 1.0 mg/mL; 24 h	Induction of apoptosis	↑ cyt-c, c-caspase-3, c-caspase-9, Bad, Bax, Noxa ↓ Bcl-2, Mcl-1, Bcl-xL, JAK/STAT3, pERK	[48]
					BALB/c mice	50 mg/kg; 14 d			
Mixture	Plant extract	Green husk	*Juglans sigillata*		EC9706, KYSE150	20, 40, 80 μg/mL; 24 h	Inhibition of proliferation, migration and survival	↑ p53, Bax, c-caspase-3 ↓ cyclin D1, MMP2, MMP9, Bcl-2	[49]
Mixture	Plant extract	Marsdenia tenacissima		*Marsdenia tenacissima (Roxb.) Moon (Asclepiadaceae)*	KYSE150, EC109	10, 20, 40, 80 mg/mL; 48 h	Inhibition of human esophageal cancer cell proliferation	↓ MAPK, ERK, JNK, p38, p-JNK, cyclin D1	[50]
Mixture	Plant extract	*Paris polyphylla* Smith (Liliaceae)			EC109	25 µg/mL, 50 µg/mL, 100 µg/mL, 200 µg/mL; 24 h	Inhibition of cell proliferation and growth	↑ connexin 26, Bad ↓ Bcl-2	[51]
Mixture	Fruit extract	Polyphenolic anthocyanin	Black raspberry		F344 rats	6.1%; 5 w	Inhibition of inflammation and tumorigenesis	↓ NF-_K_B, PTX3, sEH, COX-2	[52]
Mixture	Plant extract	Rhizome extract	*Curcuma zedoaria*		TE8, HET1A	125, 250, 500 μg/mL; 12 h (TE-8), 24 h (HET-1A)	Inhibition of angiogenesis, metastasis, and apoptosis	↑ c-caspase-9, c-caspase-3, PARP, PTEN ↓ MMP2, Akt, FGFR1, Bcl-2, STAT3	[53]
					BALB/c mice	5 mg; 30 d			
Mixture	Plant extract	Sutherlandia frutescens, Sutherlandia tomentosa	Sutherlandia		SNO	2.5, 5 mg/mL; 24 h	Induction of apoptosis	↑ c-caspase 3, c-caspase-7	[54]
Mixture	Decoction	Daikenchuto	*Ginseng Radix,* *Zanthoxylum pipericum, Zingiber officinale,* Koi		KYSE520, KYSE790	20 µg/ mL; 24, 48, 72 h	Inhibition of esophageal tumor growth and induction of apoptosis	↑ PAK1/cyclin D1, AKT/AR	[55]
					Nude mice	20 µg/ mL; 4 w			
Mixture	Decoction	*Rosa roxburghii Tratt* (CL), *Fagopyrum cymosum* (FR)			CaEs17	100 μg/mL; 48 h	Inhibition of tumor growth	↑ Bax ↓ Bcl-2, Ki-67	[56]
						120 μg/mL; 48 h			
Mixture	Decoction	Tonglian	*Herba Hedyotis diffusae* *Radix Rehmanniae praeparata* *Radix* *Angelicae sinensis* *Rhizoma Curcumae* *Radix Ophiopogonis* *Rhizoma Cimicifugae* *Semen Areca* *Herba Scutellariae* *Barbatae*		EC109	386 mg/L; 48 h	Inhibition of carcinogenesis	↓ IKKβ, NF-KB, TNF-α, IL-1β	[57]

ROS, reactive oxygen species; Bax, Bcl-2-associated X protein; Bcl-2, B-cell lymphoma; c-PARP, cleaved poly (DAP-ribose) polymerase; BID, Bcl-2 homology 3 interacting domain death agonist; PARP, poly (ADP-ribose) polymerase; Nrf2, nuclear factor erythroid-2-related factor 2; p21, cyclin-dependent kinase inhibitor 1; PTEN, phosphatase and tensin homolog; PI3K, phosphoinositide 3-kinase; AKT, protein kinase B; HSP60, heat shock 60 kDa chaperonin protein; HSPA8, heat shock 70kDa protein 8; HSP27, heat shock protein 27; DJ-1, parkinsonism associated deglycase; PRDX1, peroxiredoxin 1; NOB1, NIN1 binding protein 1 homolog; CAP1, cyclase associated actin cytoskeleton regulatory protein; GAPDH, glyceraldehyde 3-phosphate dehydrogenase; PHGDH, D-3-phosphoglycerate dehydrogenase; MAPRE1, microtubule-associated protein RP/EB family member 1; PDXK, pyridoxal kinase; p-Plk1, polo-like kinase 1; ALDH1A1, aldehyde dehydrogenase 1 family member A1; CD44, cell surface adhesion receptor; AZD7762, Chk1 and Chk2 kinase inhibitor; Prx I, peroxiredoxin I; p53, tumor protein p53; Cyt-c, cytochrome-c; L-NAC, N-acetyl-L-cysteine; ZO1, zonula occludens 1; Zeb1, zinc finger e-box-binding homeobox 1; Slug1, snail family transcriptional repressor 2; p-p85, phospho-PI3 kinase p85; Ki-67, marker of proliferation; IL-6, interleukin-6; CRP, C-reactive protein; COX-2, cyclooxygenase-2; PGE2, prostaglandin E2; NFKB, nuclear factor-kappa B; HDAC1, histone deacetylase 1; RELA, CDK, cyclin-dependent kinase; EGFR, epidermal growth factor receptor; CDKN1B, cyclin dependent kinase inhibitor 1B; DR5, death receptor 5; PTX, pentraxin; MAPK, mitogen-activated protein kinase; PCNA, proliferating cell nuclear antigen; RTK, receptor tyrosine kinases; Bcl-xL, B-cell lymphoma-extra-large; ERK extracellular signal-regulated kinase; AP-1, Activator protein 1; JNK, c-jun N-terminal kinase; p38, mitogen-activated protein kinase; p-JNK, phospho-c-Jun N-terminal kinase; p-p38, p38 mitogen-activated protein kinase; Bad, Bcl-2-associated death promoter; PTEN, phosphatase and tensin homolog; MMP, matrix metallopeptidase; FGFR1, fibroblast growth factor receptor 1; STAT3, signal transducer and activator of transcription 3; PGF2, prostaglandin f2 alpha; sEH, soluble epoxide hydrolase; CDC2, cell division cycle 2; Noxa, phorbol-12-myristate-13-acetate-induced protein 1; Mcl-1, myeloid cell leukemia 1; JAK/STAT3, janus kinase/signal transducer and activator of transcription 3; pERK, PKR-like ER kinase; p-p53, p53 mitogen-activated protein kinase; PAK1, p21 activated kinase 1; IKKβ, inhibitor of nuclear factor kappa-B kinase subunit beta; TNF-α, tumor necrosis factor alpha; IL-1β, interleukin 1 beta; h, hours; d, days; ↑, upregulation; ↓, downregulation.

### 2.2. Anti-Angiogenesis and Natural Products

Aggregate fruits such as blackberry and black raspberry possessed anti-angiogenic properties which came into effect when exposed to EC cells. Moreover, extracts and decoctions previously mentioned that possess apoptotic functions also prevented angiogenesis (Table 2).

Hadisaputri et al. reported that rhizome extract from *Curcuma zedoaria* (CZ) has anti-angiogenic, anti-metastatic, and apoptotic effecTs on esophageal carcinoma cells [53]. The treatment of CZ extract in both TE-8 and HET-1A cells activated cleaved caspase-9, -3, cleaved PARP, and PTEN and deactivated Bcl-2, MMP-2, AKT, FGFR1, and STAT3. CZ extract subcutaneously injected into BALB/c mice has been found to contain suitable constituents that modulate multi-targets against proteins in esophageal tumor cells.

Medda et al. reported the anti-inflammatory and anti-angiogenic properties of black raspberry extract (BRE) on HEMEC (esophageal squamous cancer cell) [59]. HEMEC cell lines exposed to BRE exhibited the attenuation of gene proteins ICAM-1 and VCAM-1. In addition, the activation of COX-2, PGE2, and NF-_K_B proteins increased. Regarding anti-inflammatory and anti-angiogenic effects, BRE could be an organ-specific novel remedy.

Huang et al. reported black raspberry (BRB) as a novel remedy for esophageal cancer, containing chemopreventive effects and, ultimately, a tumor suppressing agent [60]. F344 rats were orally fed diets containing 5% BRB. BRB reduced methylation in 81 regions/genes, along with the demethylation of Sfrp4 regions, a specifically methylated protein for esophageal squamous cancer cells and adenocarcinoma, evidenced by dysplastic lesions in the cancer cells.

Wang et al. demonstrated that black raspberry (BRB) has anti-inflammatory, apoptotic, and anti-angiogenic effects in esophageal carcinogenesis [46]. A diet containing 5% BRB powder was given to F344 rats. BRB influenced the COX pathway of the arachidonic acid metabolism by downregulating COX-2 and PGF2 levels. In addition, BRBs were found to affect the LOX pathways of the arachidonic acid metabolism through the expression of MMP10, inhibiting LTB4. The study also reported that the expression of genes can be influenced by BRB during the late stages of rat esophageal cancer.

Shi et al. reported the anti-angiogenic effects of Aidi in esophageal cancer [61]. Aidi managed to reduce the migration span of EC cells by the reduction in visible capillary-like tube networks in tumor blood vessels. In addition, EMT signaling was inhibited, evidenced by the increased activation of cadherin-1. Nude mice were intraperitoneally injected with Aidi, which showed the attenuated activity of vimentin and VEGF-A, while the activity of cadherin-1 was encouraged.

**Figure 2:**
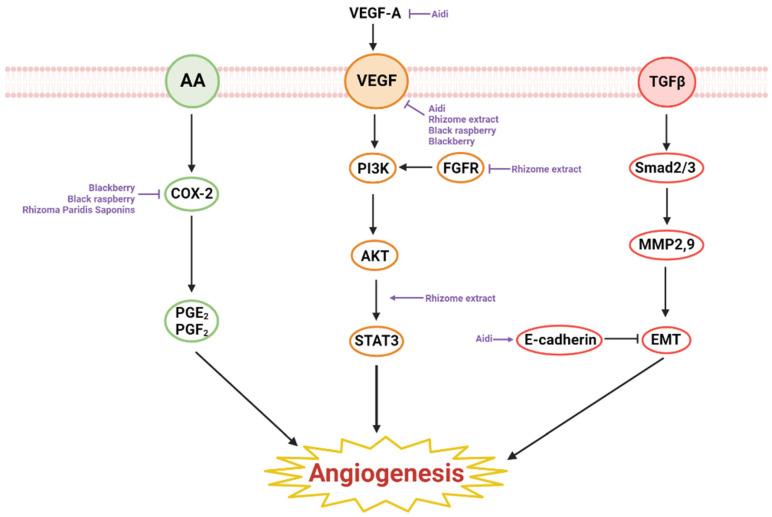
Schematic diagram of angiogenesis-inhibiting natural products in esophageal cancer.

The inhibitory effect being therapeutic in cancer, angiogenesis is the growth of blood vessels from an already existing one. Five studies examined the anti-angiogenic effects of natural products in esophageal cancer. Most of the compounds or extracts that had anti-angiogenic properties on esophageal cancer were fruits. An extract and a decoction were also found to have apoptotic properties on esophageal cancer. Esophageal cancer cell lines used for studies regarding anti-angiogenic effects in vitro were TE8, HET1A, HEMEC, EC9706, and KYSE70. BALB/c mice and F344 rats were mainly used as animal models for research in vivo. Within anti-angiogenic natural products, blackberry lacked cytotoxicity testing and the rest had little to no cytotoxicity in normal cells [59]. Studies regarding black raspberry and Aidi only conducted in vitro experiments; therefore, there is a need for further examination of their anti-angiogenic effects in mice models [46,60,61]. Blackberry was only researched using vivo experiment, and thus, in vitro experiments are required for further investigation [59]. In addition to their anti-angiogenic effects, some substances induced apoptosis, anti-metastasis, and anti-inflammation effects on esophageal cancer. The anti-angiogenic mechanisms of the natural product are elucidated in Figure 2.

**Table 2:** Anti-angiogenesis-related natural products.

Classification	Compound/Extract	Source	Experimental Model	Dose; Duration	Efficacy	Mechanism	Reference
Plant extract	Rhizome extract	*Curcuma zedoaria*	TE8, HET1A	125, 250, 500 μg/mL; 12 h (TE-8), 24 h (HET-1A)	Inhibition of angiogenesis, metastasis, and apoptosis	↑ c-caspase-9, c-caspase-3, PARP, PTEN ↓ MMP-2, Akt, FGFR1, Bcl-2, STAT3	[53]
			BALB/c mice	5 mg; 30 d			
Fruit extract	Blackberry		NMBA, F344 rats	5%; 5 w	Inhibition of tumorigenesis through epigenetic regulation	↓ Sfrp4, DNMT3B, DNMT1, b-catenin, iNOS, COX-2, NF-_K_B, pS6	[59]
Fruit extract	Black raspberry		HEMEC	100 μg/mL; 2 h	Inhibition of inflammation and angiogenesis	↓ COX-2, PGE_2_, VEGF, ICAM-1, VCAM-1, NF-_K_B	[60]
Fruit extract	Black raspberry		F344 rats	5%; 35 w	Inhibition of inflammation, apoptosis, and angiogenesis	↓ MMP10, COX-2, PGF2	[46]
Decoction	Aidi	*Astragalus membranaceus Acanthopanax Mylabris*	EC9706, KYSE70	1.5, 3, 6, 12, 24, 48, 96 mg/mL; 24, 48 h	Inhibition of epithelial-mesenchymal transition and angiogenesis	↑ cadherin-1 ↓ VEGF-A, vimentin, cadherin-2	[61]
			BALB/c mice	1, 2, 4 g/kg; 15 d			

PARP, poly (ADP-ribose) polymerase; PTEN, phosphatase and tensin homolog; MMP, matrix metallopeptidase; AKT, protein kinase B; FGFR1, fibroblast growth factor receptor 1; Bcl-2, B-cell lymphoma; STAT3, signal transducer and activator of transcription 3; Sfrp4, secreted frizzled-related protein 4; DNMT3B, DNA methyltransferase 3 beta; DNMT1, DNA (cytosine-5)-methyltransferase 1; iNOS, nitric oxide synthase; COX-2, cyclooxygenase-2; NF-kB, nuclear factor-kappa B; pS6, phospho-S6; PGE2, prostaglandin E2; VEGF, vascular endothelial growth factor; ICAM-1, intercellular adhesion molecule 1; PGF2, prostaglandin f2 alpha; VCAM-1, vascular cell adhesion molecule 1; VEGF-A, vascular endothelial growth factor A; h, hours; d, days; w, weeks; ↑, upregulation; ↓, downregulation.

### 2.3. Anti-Metastasis and Natural Products

Natural compounds, fungi, proteins, extracts, and decoctions often used in TCM and Kampo medicine were also found to have anti-metastatic effects on EC cells (Table 3).

Xu et al. reported synephrine’s (SYN) anti-metastatic potentials on EC cells [62]. SYN inhibited EC cell-initiated cell migration and invasion through the upregulation of E-cadherin and downregulation of vimentin. The apoptotic and anti-proliferative effects were found through the weakening of AKT/ERK signaling by the attenuation of Galectin-3. In vivo, mice saw a decrease in tumor size of 71.6% by the downregulation of Galectin-3 and ultimately the inactivation of the AKT/ERK cascade.

Wang et al. proposed that garcinol (GAR) contains metastatic effects made possible through its control of significant proteins [63]. KYSE150 exposed to GAR exhibited a vast reduction in cell migration. GAR especially negatively regulated proteins p300 and p-Smad2/3, along with TGF-β1. In vivo, mice were intraperitoneal injected with GAR and a proliferation marker, Ki-67, decreased. Tumor nodules decreased, along with the weight, when followed up with the 5-FU treatment.

Gu et al. reported the inhibitory effects of icariin (ICA) on esophageal squamous cancer cell metastasis [64]. ICA presented its dominant effect on both cell migration and fold cell invasion, significantly weakening the metastasis-prone EC cells. ICA also inhibited anchorage-independent esophageal cancer cell growth. In vivo, ICA exposure resulted in a drastic anti-metastatic effect from its upregulation of ROS and downregulation of the PI3K/AKT signaling pathway and the proteins associated with it.

Li et al. reported the anti-metastatic effects of the combination of lactoferrin (LF) and linolenic acid (LA) on esophageal squamous cancer cells [65]. KYSE450 cells exposed to LF and LA had significant viability inhibitions. Mice were subcutaneously injected with 50 mg/kg of LF and 5 mg/kg of LA. This resulted in the inhibition of tumor metastasis by the inhibition of the JAK2/STAT3/ERK/AKT pathway by the downregulation of the proteins involved in activating the aforementioned pathway.

Yan et al. reported the use of gypenoside (Gyp) as an anti-metastatic remedy for esophageal cancer cells [66]. Cell proliferation was restrained in EC109 cells with exposure to Gyp, decreasing esophageal squamous cancer cell viability. Time-dependently, esophageal squamous cancer cell membranes were damaged, along with morphological changes, which included transformations into circular structures. ROS generation increased dose-accordingly, but the opposite resulted using high doses of Gyp going above 150 μg/mL.

Ding et al. reported griffipavixanthone’s (GPX) anti-metastatic properties [67]. GPX showed inhibitory effects on cell migration and invasion in KYSE70 and TE1 cell lines. GPX also inhibited proliferation through cell cycle arrest at the G2/M phase. In nude mice, intraperitoneally injected GPX attenuated pulmonary metastasis. Proteins phospho-ERK and Ki-67 were significantly suppressed, resulting in metastasis in the liver, kidney, heart, and spleen infringing upon the RAS-RAF-MEK-ERK cascade-involved proteins and cyclinB1 levels.

Batirel et al. reported the anti-metastatic effects of walnut oil (WO) specifically on esophageal adenocarcinoma cells [68]. Esophageal adenocarcinoma cells lost their mobility with exposure to WO, resulting in reduced cell migration and invasion. The adhesion strength of these cancer cells was evidently reduced, along with colony formation. Cell cycle was arrested at the G0/G1 phase by the decrease in NF-_K_B protein activity, which caused necrosis in esophageal squamous cells.

Hadisaputri et al. reported that rhizome extract from Curcuma zedoaria (CZ) has anti-metastatic effects on esophageal carcinoma cells [53]. The treatment of CZ extract in TE-8 and HET-1A cells activated cleaved caspase-9, cleaved caspase-3, PARP, and PTEN while deactivating Bcl-2, MMP-2, AKT, FGFR1, and STAT3. In addition, CZ subcutaneously injected into BALB/c mice showed constructive evidence regarding the anti-metastatic potential of CZ.

Liu et al. reported the inhibition of epithelial-mesenchymal migration Antrodia cinnamomea mycelial fermentation broth (AC-MFB) has on esophageal squamous cancer cells [69]. AC-MFB exhibited properties causing a vast reduction in cell viability. AC-MFB was used with TGF-β, which resulted in the upregulation of E-cadherin, making it clear that AC-MFB reduces esophageal cancer cell viability. In addition, EMT specifically turned on by TGF- β initiated twist.

Li et al. reported the use of Andrographis paniculata (AP) as a novel remedy for esophageal squamous cell carcinoma for its anti-metastatic effects [70]. The exposure of AP on EC109 cells resulted in a 61% attenuation in cell migration and invasion. In addition, expressions of metastasis-related genes TM4SF3 and MMP9 significantly decreased. In vivo, mice were intraperitoneally injected with AP, resulting in a reduced size of the esophageal tumor, along with the attenuation of micro-metastasis to the lungs and liver.

Li et al. demonstrated the restricting effects of green husk on esophageal squamous cancer cell lines [49]. Green husk extractives caused apoptosis, reduction in proliferation, and migration. The EtOH extract, EtOAc soluble fraction, and GA extractives impaired the motility and invasion of cancer cells by prohibiting migration and proliferation by the downregulation of MMP2, MMP9, cleaved caspase-3, Bcl-2, and the upregulation of Bax. Cell cycle arrest was initiated through the attenuation of cyclin D1.

Xie et al. reported purpurogallin’s (PPG) anti-metastatic effects on esophageal squamous cancer cells [71]. KYSE EC cells exposed to PPG resulted in the inhibition of anchorage-dependent tumor cells and anchorage-independent cell growth. Female mice were orally administered with PPG, exhibiting significant reduction in phosphorylation of protein MEK1/2. This contributed to the downregulation of protein ERK1/2, resulting in the inhibition of the MEK/ERK signaling pathway.

Shi et al. reported the use of qigesan (QGS) as a remedy for esophageal squamous cancer cell migration and invasion [72]. QGS at a dose of 100 μg/mL for 24 h resulted in a reduced number of invading cells in human esophageal cancer cells. In addition, QGS improved signaling among neighboring cells by strengthening the gap junction, thus weakening the invasion of cancer cells by accreting connexins 26 and 43.

Kong et al. reported the use of qigesan (QGS) decoction for its force against invasion and metastasis of EC cells [73]. Cell migration significantly decreased, and invasion abilities were inhibited, resulting in a decrease in the number of EC cells passing through the Transwell chamber. The expressions of proteins involved in the Gas6/Axl and PI3K/AKT and NF-_K_B signaling pathways, along with the MMP2 and MMP9 protein pathway, were reduced, downregulating the metastatic qualities of EC cells.

**Figure 3:**
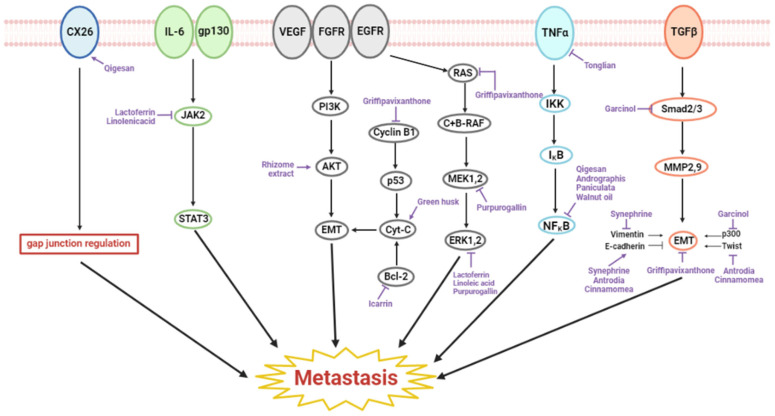
Schematic diagram of metastasis-inhibiting natural products in esophageal cancer.

Metastasis, one of the most fatal characteristics of cancer, is when cancer cells spread from the point of origin to distant areas of the body through lymph vessels or bloodstream. In total, fourteen substances had anti-metastatic effects on esophageal cancer. Most of the natural products that had anti-metastatic properties on esophageal cancer were organic compounds found in plants. Besides organic compounds, extracts, fruits, decoctions fungi, and plants were also found to have metastatic effects on esophageal carcinoma. The esophageal cancer cell lines that were frequently studied regarding their anti-metastatic effects in vitro were KYSE150, KYSE450, EC109, and TE1. BALB/c mice were predominantly used as the animal model in vivo. All natural products containing anti-metastatic effects had little to no cytotoxicity in normal cells. Gypenoside, walnut oil, *Antrodia cinnamomea*, green husk, and Qigesan were only researched in in vitro experiments, and thus, additional investigation into the anti-metastatic effects in mice models is required [49,66,68,69,72]. In vitro study cannot fully represent the whole interaction between the esophageal cancer cell and the natural substances listed and, thus, in vivo experiments are required. The rest of the research performed both in vivo and in vitro studies. Besides the anti-metastatic effects, some substances also inhibited cancer growth. A few of them were also effective in preventing angiogenesis and apoptosis. The anti-metastatic mechanisms of the natural product are elucidated in Figure 3.

**Table 3:** Anti-metastasis-related natural products.

Classification	Compound/Extract	Source	Experimental Model	Dose; Duration	Efficacy	Mechanism	Reference
Alkaloid	Synephrine	Citrus	KYSE30, KYSE270	5, 10 µM; 24 h	Inhibition of growth and metastasis	↑ E-cadherin ↓ vimentin, Galectin-3, AKT/ERK	[62]
			BALB/c mice	10, 20 mg/kg; 30 d			
Benzophenone	Garcinol	*Garcinia yunnanensis* Hu	KYSE150, KYSE450	5, 10, 15 μM; 24 h	Suppression of metastasis	↑ E-cadherin ↓ TGF-β1, p-300, p-Smad2/3	[63]
			BALC/c mice	20 mg/kg; 5 w			
Flavonoid	Icariin	*Epimedium* spp.	KYSE70	20, 40, 80 μM; 48 h	Inhibition of growth and metastasis	↓ AKT, STAT3 ↓ PI3K/AKT, ROS	[64]
			immunodeficient mice	40 μg/g; 4 w			
Protein, fatty acid	lactoferrin, linolenic acid	Transferrin, omega-3 fatty acid	KYSE450	0.01, 0.05, 0.1, 0.5, 1, 5 g/L; 48 h	Inhibition of metastasis	↓ JAK2, STAT3, ERK, AKT, LCT	[65]
			BALB/c mice	55 (LF-50; LA-5) mg/kg; 24 d			
Triterpenoid	Gypenoside	*Gynostemma pentaphyllum* Makino	EC109	50, 100, 150, 200 µg/mg; 24, 48, 72 h	Inhibition of proliferation and migration	↑ ROS	[66]
Xanthone	Griffipavixanthone	*Garcinia yunnanensis* Hu	TE1, KYSE150	5, 10, 15, 20 μM; 48 h	Inhibition of tumor metastasis and proliferation	↓ RAF-MAPK, cyclin B1, EMT	[67]
			nude mice	20 mg/kg; 2 d			
Plant extract	Walnut oil	*Juglans regia* L.	OE19	10, 20, 30, 40 mg/mL; 24 h	Inhibition of migration	↓ NF-_K_B, cyclin B1	[68]
Plant extract	Rhizome extract	*Curcuma* zedoaria	TE8, HET1A	125, 250, 500 μg/mL; 12 h (TE-8), 24 h (HET-1A)	Inhibition of angiogenesis, metastasis, and apoptosis	↑ caspase-9, caspase-3, PARP, PTEN ↓ MMP-2, Akt, FGFR1, Bcl-2, STAT3	[53]
			BALB/c mice	5 mg; 30 d			
Plant extract	*Antrodia cinnamomea*	*Cinnamomum kanehirai*	BE3	1.0 mg/mL; 24 h, 48 h, 72 h	Inhibition of epithelial to mesenchymal transition	↑ Zeb1 ↓ HIF-1α, E-cadherin, Twist	[69]
Plant extract	*Andrographis paniculata*	Acanthaceae	EC109	800, 1600, 3200 μg/mL; 24 h	Inhibition of metastasis	↓ TM4SF3, HER2, CXCR4, MMP2, p-NF-_K_B, MMP9	[70]
			BALB/c mice	1600 mg/kg; 3 w			
Plant extract	Green husk	Juglans sigillata	EC9706, KYSE150	20, 40, 80 μg/mL; 24 h	Inhibition of proliferation, migration and survival	↑ p53, p-p53, Bax, c-caspase-3 ↓ cyclin D1, MMP2, MMP9, Bcl-2	[49]
Plant extract	Purpurogallin	Nutgalls, oak bark	KYSE30, KYSE70, KYSE410, KYSE450, KYSE510	5, 10, 20, 40, 60 µM; 48 h	Inhibition of metastasis	↓ ERK1, ERK2, MEK1, MEK2	[71]
			Nude mice	100 mg/kg; 2 weeks			
Decoction	Qigesan	*Radix Curcumae* *Radix Glehniae* *Radix Salviae Miltiorrhizae* *Bulbus Fritillaria Thunbergii* *Glabrous Greenbrier Rhizome* *Villous Amomrum Fruit* *Lotus Leaf* *Pinellia Tuber* *Blighted Wheat* *Radix Asparagi* *Rhizoma Dioscoreae*	TE1, TE13, EC109	1, 3, 10, 30, 50, 100 μg/mL; 24 h	Inhibition of migration and invasion	↑ Cx26, Cx43	[72]
Decoction	Qigesan	*Curcuma wenyujin* *Adenophora tetraphylla* *Radix Salviae Miltiorrhizae* *Fritillaria Thunbergii Bulbus* *Poria* *Amomum villosum Lour* *Lotus Leaf* *Pinellia ternate* *Blighted wheat* *Radix asparagi* *Dioscorea opposita Thunb*	EC109, TE1	100, 200 μg/mL; 24 h	Reduction in invasion and metastasis	↓ Gas6, Axl, PI3K, AKT, NF-_K_B	[73]

p-AKT, phospho- protein kinase B; p-ERK, phospho-extracellular-regulated kinase; AKT, protein kinase B; ERK, extracellular-signal-regulated kinase; TGF-β1, transforming growth factor-β1; p-300, E1A associated protein p300; p-Smad2/3, phospho-Smad2/3 antibody; p-Plk1, polo-like kinase 1; STAT3, signal transducer and activator of transcription 3; PI3K, phosphoinositide 3-kinase; ROS, reactive oxygen species; JAK2, janus kinase 2, LCT, lithocholyltaurine; RAF-MAPK, rapidly accelerated fibrosarcoma mitogen-activated protein kinase; EMT, epithelial-mesenchymal transition; NF-_K_B, nuclear factor-kappa B; PARP, poly (ADP-ribose) polymerase; PTEN, phosphatase and tensin homolog; MMP, matrix metallopeptidase; FGFR1, fibroblast growth factor receptor 1; Bcl-2, B-cell lymphoma; Zeb1, zinc finger e-box-binding homeobox 1; HIF-1α, hypoxia-inducible factor 1-alpha; TM4SF3, transmembrane 4 superfamily 3; HER2, human epidermal growth factor receptor 2; CXCR4, C-X-C motif chemokine receptor 4; p-NF-_K_B, phospho- nuclear factor-kappa B; p53, tumor protein; p-p53, p53 mitogen-activated protein kinase; Bax, Bcl-2-associated X protein; MEK, mitogen-activated protein kinase kinase; Cx26/43, connexin 26, 43; Gas6, growth arrest-specific gene; Axl, tyrosine-protein kinase receptor UFO; PI3K, phosphoinositide 3-kinase; h, hours; d, days; w, weeks; ↑, upregulation; ↓, downregulation.

### 2.4. Anti-Resistance and Natural Products

When cancer patients go through radiotherapies or medications, their bodies resist certain drugs meant to target cancer cells, preventing effective cancer treatments. Natural products, alkaloids, and terpenes, prevent this resistance in EC cells (Table 4).

Wang et al. reported the use of tetrandrine (TET) as a suitable treatment in inhibiting drug-resistant esophageal cancer cells [74]. Cisplatin-resistant esophageal cancer cells exposed to TET exhibited an active apoptotic behavior. TET reversed cytotoxicity by attenuating the expression of ABC transporters, especially MRP1. In addition, intracellular CMFDA accumulation indicates drug-sensitivity in cancer cells and TET treatment.

Meng et al. presented the combination therapy of natural borneol (NB) and paclitaxel (PTX) for treating EC cells as they enhance PTX-induced apoptosis through their synergistic effects [42]. PTX alone exhibited its anti-metastatic effects 6 h after exposure. With the addition of NB, esophageal squamous cancer cells lost their viability within 3 h. A longer exposure to PTX paired with a larger portion of NB showed significant anti-cancer effects through the activation of c-caspase-3 and downregulation of AKT.

**Figure 4:**
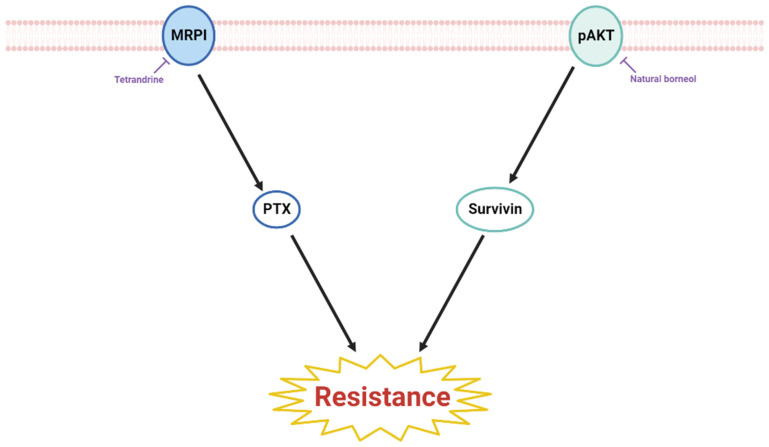
Schematic diagram of resistance-sensitizing natural products in esophageal cancer.

Drug resistance in cancer, one of the main causes of the failure of treatment, is when cancer cells become resistant to chemotherapies. Two substances were found to inhibit drug resistance on esophageal cancer. The natural products that induced anti-resistance on esophageal cancer were organic compounds. Esophageal cancer cell lines used for research regarding anti-resistance in vitro were YES2, DDP, HET1A, TE1, and TE13. There were no animal models used in this category of research. Within natural products that have anti-resistance effects on esophageal cancer, all studies conducted cytotoxicity tests and found that they have little to no toxicity in normal cells [42,74]. However, both areas need in vivo experiments for further examination. Among the natural products listed, tetrandrine reversed drug resistance by regulating MRP1, thus showing its potential to be an adjunct to clinical chemotherapy on esophageal cancer [42,74]. The anti-resistance mechanisms of the natural product are elucidated in Figure 4.

**Table 4:** Anti-resistance-related natural products.

Classification	Compound/Extract	Source	Experimental Model	Dose; Duration	Efficacy	Mechanism	Reference
Alkaloid	Tetrandrine	*Radix Stephania tetrandra* S. Moore	YES2/DDP	0.1, 0.3, 1, 3, 10, 30 µM; 72 h	Reduction in drug resistance	↓ MRP1	[74]
Terpene	Natural Borneol	camphor	HET1A, TE1, TE13	20, 40, 80 μg/mL; 24, 48, 72 h	Enhancement of paclitaxel-induced apoptosis	↑ caspase-3, PTX ↓ AKT, survivin	[42]

MRP1, multidrug resistance-associated protein 1; PTX, pertussis toxin; AKT, protein kinase B; h, hours; ↑, upregulation; ↓, downregulation.

### 2.5. Clinical Trials and Natural Products

Two clinical trials regarding green tea and *Brucea javanica* (L.) Merr. were conducted on esophageal cancer patients. Both clinical trials were conducted on the efficacies of each compound extract on the prevention of EC through single or paired treatments with radiotherapy (Table 5).

Lightdale et al. presented a clinical trial of the efficacy of green tea catechin (GTC) extract in preventing esophageal squamous cell carcinoma in patients with Barrett’s esophagus [75]. Fifty-five patients with grade I of Barrett’s esophagus were randomly assigned to four different groups. Group 1 orally took one capsule of GTC extract for 6 months. Group 2 took two capsules, group 3 took 3three and lastly, group 4 took one to three capsules of placebo. The results of this clinical experience have not been posted due to unknown reasons.

Shan et al. reported a clinical trial of the synergistic effects of oral Fructus bruceae oil combined with radiotherapy in esophageal squamous cancer cells [76]. Eighty patients diagnosed with grade II-III squamous cell carcinoma were randomly divided into two groups. More than half (21) of the patients that were pair-treated (Group A) experienced complete remission, while only one-fourth of the patients that only went through radiotherapy (Group B) did so. Pair-treatment also improved esophageal obstruction caused by esophageal squamous cancer cells.

Two clinical trials were found for treating esophageal cancer with natural products. Research on the clinical effects of green tea on EC was conducted in the US and first published in 2005 and last updated in 2014. Research regarding the natural product *Brucea javanica* (L.) Merr. was performed in China and published in 2011. This study on green tea was conducted in patients with grade I of EC, according to the esophageal cancer staging criteria. The other research was conducted grade II and III patients. Both studies were completed, and one of them published their results. Both studies recruited more than 50 participants. The natural products were administered orally in both studies. In these two studies, *Brucea javanica* (L.) Merr. combined with radiotherapy showed promising results in regulating esophageal cancer and relieving symptoms of dysphagia, the most common symptom of esophageal cancer.

**Table 5:** Clinical trials.

Compound/Extract	Source	Phase	Patients	Status	Efficacy	Reference
Catechin extract	green tea	I	55	Completed	Efficacy of green tea catechin extract for prevention of esophageal cancer	[77]
Fructus bruceae	*Brucea javanica* (L.) Merr.	II, III	80	Completed	Efficacy combined with radiotherapy for the treatment of esophageal cancer	[78]

## 3. Discussion

Apoptosis is a programmed cell death [15]. Usually, cells that find themselves failing to function according to changes in body conditions turn on self-cell death. However, most of those that do not recognize their failure soon aggregate into malignantly functioning cells that do not go through self-cell death and form cancer [16]. According to the NIH, all cancers have a tendency to metastasize, where cancer cells break away from the primary location and go on to secondary and tertiary locations [13]. Metastatic cancer occurs when cancer cells migrate through new blood vessels, a process called angiogenesis. According to the American Cancer Society, doctors claim that metastasized cancers look exactly like the ones from the esophagus [77]. This means esophageal cancer could possibly be one of the cancers which most easily spreads to the general body in a matter of months. Numerous types of surgeries and drugs available in combatting EC are suggested by the American Cancer Society. However, therapeutics do not always work on cells that are malignant [78]. This is because cancer cells grow resistant towards certain drugs to prevent their metastasis and angiogenesis, inevitably making drugs a mediocre cure for EC [79]. The mutational basis for drug resistance is caused by drug inactivation, drug target alteration, drug efflux, DNA damage repair, cell death inhibition, epithelial-mesenchymal transition, inherent cell heterogeneity, and epigenetic effects [78]. In addition, the NIH claims that the adverse effects from chemotherapy are unavoidable and exempt them from being actively utilized because they reduce the patients’ quality of life [80]. Some of the side effects include vomiting, nausea, fever, perforations, severe infection from within, diarrhea, constipation, and allergic reactions [10]. Thus, this calls for natural products that can be consumed by esophageal cancer patients. It is crucial to provide people with an easier approach to cure EC, the eighth most commonly occurring cancer in the world, through natural products [81].

In this systematic review, the anti-cancer activity of natural products on esophageal cancer was scrutinized. The classification, source, experimental model, dose, duration, efficacy, and mechanism of each of the studies regarding different natural products were markedly categorized. In total, five tables were assembled based on the anti-cancer mechanisms of the natural products. The systematic reviews published within the last 10 years regarding the effects of natural products on esophageal cancer were mainly conducted in conjunction with radiotherapy or chemotherapy. Existing reviews focused more on the effects of natural products on the adverse symptoms caused by chemotherapy. All studies used randomized controlled trials and, particularly, Chen et al. focused only on clinical trials conducted in China. There were no systematic reviews found that solely examined the association between esophageal cancer and natural products. However, in this paper, all of the studies found which discussed the intrinsic efficacies of natural products were analyzed by forming five different categories comprised of cancer mechanisms and clinical trials. In particular, studies regarding pristimerin, which downregulates CDKN1B; matrine, which activates the Bid-mediated mitochondrial pathway; Fructus forsythia, which activates Bcl-2 and downregulates JAK/STAT3 and ERK signaling pathways; and lactoferrin, which inactivates the phosphorylation of JAK2/STAT3/Erk/AKT proteins were administrated at a low concentration of 1 mg/mL or less, and both in vivo and in vitro studies were conducted, illustrating their potential to be utilized as lead compounds in treating esophageal cancer.

The evaluation of studies published only in English and the shortage in the number of clinical trials and anti-resistance-related research are the main drawbacks of this study. Extensive research is required through utilizing additional search engines other than databases such as PubMed and Google Scholar in order to achieve wide-ranging and accurate data collection by including studies published in other languages. Thirty-seven studies regarding apoptosis, five studies regarding angiogenesis, and fourteen studies regarding metastasis were found. However, two categories, clinical trial and anti-resistance, had only two studies each on esophageal cancer and natural products, which means there is a lower number of research works available regarding these areas. Moreover, some of the studies lacked in vitro or in vivo studies, meaning further examination is required in order to clearly elucidate the effects of natural products on esophageal cancer. Taking these aspects into consideration, comprehensive studies on the anti-esophageal cancer effects of natural substances are advised to be conducted. Despite these limitations, this systematic review offers an overview of the effects of natural products on esophageal cancer. Thus, natural products analyzed in this study have the potential to provide therapeutic benefits regarding esophageal cancer.

## 4. Conclusions

This study reviewed the anti-esophageal cancer effects of natural products by classifying fifty-seven studies into various anti-cancer mechanisms. The regulatory factors that encourage anti-cancer activity were summarized for an overview of the effects of natural products. Natural products are promising as they have the potential to treat esophageal cancer. Therefore, additional in vitro, in vivo, and clinical studies should be conducted in order to fully utilize these promising resources.

## Abbreviations

AA	arachidonic acid
AKT	protein kinase B
Bax	Bcl-2-associated X protein
Bcl-2	B-cell lymphoma-2
Bid	Bcl-2 homology 3 interacting domain death agonist
c-PARP	cleaved poly (DAP-ribose) polymerase
RAF	Rapidly Accelerated Fibrosarcoma
CD44	cell surface adhesion receptor
CDC2	cell division cycle 2
CDK2	cyclin-dependent kinase 2
CDK4	cyclin-dependent kinase 4
CDK6	cyclin-dependent kinase 6
COX-2	cyclooxygenase-2
CX26	connexin 26
Cyt-c	cytochrome-c
EGFR	epidermal growth factor receptor
EMT	epithelial-mesenchymal transition
ERK	extracellular signal-regulated kinase
FGFR	fibroblast growth factor receptor
gp130	Glycoprotein 130
HDACI	histone deacetylase 1
I_K_B	inhibitor of kappa B
IKK	inhibitor of kappa B kinase
IL-6	interleukin-6
JAK	janus kinase
JNK	c-jun N-terminal kinase
MAPK	mitogen-activated protein kinase
MEK 1	mitogen-activated protein kinase kinase 1
MEK 2	mitogen-activated protein kinase kinase 2
MMP 2	Matrix metallopeptidase 2
MMP 9	Matrix metallopeptidase 9
MRPI	multidrug resistance-associated protein 1
mTOR	mammalian target of rapamycin
NF_K_B	nuclear factor kappa B
pAKT	Phospho protein kinase B
PGE_2_	prostaglandin E2
PGF_2_	prostaglandin f2
PI3K	phosphoinositide 3-kinase
PIKI	polo-like kinase 1
PTX	pertussis toxin
RAS	Renin angiotensin system
ROS	reactive oxygen species
STAT3	signal transducer and activator of transcription 3
TGFβ	transforming growth factor-β
TNFα	tumor necrosis factor alpha
VEGF	vascular endothelial growth factor

## References

[1] National Cancer Institute (2021). Esophageal Cancer Treatment. https://www.cancer.gov/types/esophageal/patient/esophageal-treatment-pdq#_107.

[2] National Cancer Institute Esophageal Cancer. https://www.cancer.gov/types/esophageal.

[3] International Agency for Research on Cancer (2020). Oesophagus. https://gco.iarc.fr/today/data/factsheets/cancers/6-Oesophagus-fact-sheet.pdf.

[4] Sung H., Ferlay J., Siegel R.L., Laversanne M., Soerjomataram I., Jemal A., Bray F. (2021). Global Cancer Statistics 2020: GLOBOCAN Estimates of Incidence and Mortality Worldwide for 36 Cancers in 185 Countries. CA Cancer J. Clin..

[5] Cancer.Net (2021). Esophageal Cancer: Types of Treatment. https://www.cancer.net/cancer-types/esophageal-cancer/types-treatment.

[6] American Cancer Society (2021). Targeted Drug Therapy for Esophageal Cancer. https://www.cancer.org/cancer/esophagus-cancer/treating/targeted-therapy.html.

[7] Gordon M.A., Gundacker H.M., Benedetti J., Macdonald J.S., Baranda J.C., Levin W.J., Blanke C.D., Elatre W., Weng P., Zhou J.Y. (2013). Assessment of HER2 gene amplification in adenocarcinomas of the stomach or gastroesophageal junction in the INT-0116/SWOG9008 clinical trial. Ann. Oncol..

[8] Sasaki T., Hiroki K., Yamashita Y. (2013). The role of epidermal growth factor receptor in cancer metastasis and microenvironment. BioMed Res. Int..

[9] Hoeben A., Landuyt B., Highley M.S., Wildiers H., Van Oosterom A.T., De Bruijn E.A. (2004). Vascular endothelial growth factor and angiogenesis. Pharmacol. Rev..

[10] Ueki T., Fujimoto J., Suzuki T., Yamamoto H., Okamoto E. (1997). Expression of hepatocyte growth factor and its receptor c-met proto-oncogene in hepatocellular carcinoma. Hepatology.

[11] Weill Cornell Medcine Newsroom (2020). Immunotherapy Extends Survival in Esophageal Cancer. https://news.weill.cornell.edu/news/2020/12/immunotherapy-extends-survival-in-esophageal-cancer.

[12] Niu J., Gelbspan D., Weitz D., Markman M., Quan W. (2014). HER2-positive, trastuzumab-resistant metastatic esophageal cancer presenting with brain metastasis after durable response to dual HER2 blockade: A case report. J. Gastrointest. Oncol..

[13] National Cancer Institute Metastasis. https://www.cancer.gov/publications/dictionaries/cancer-terms/def/metastasis.

[14] National Cancer Institute (2016). Why Do Cancer Treatments Stop Working? Overcoming Treatment Resistance. https://www.cancer.gov/about-cancer/treatment/research/drug-combo-resistance.

[15] Elmore S. (2007). Apoptosis: A review of programmed cell death. Toxicol. Pathol..

[16] Cleveland Clinic (2021). Metastatic Cancer. https://my.clevelandclinic.org/health/diseases/17224-metastatic-cancer.

[17] Islam M.T. (2018). A literature-based phytochemical evidence and biological activities of Trichosanthes dioica Roxb. Orient. Pharm. Exp. Med..

[18] Sun L., Zhao W., Li J., Tse L.A., Xing X., Lin S., Zhao J., Ren Z., Zhang C.X., Liu X. (2021). Dietary flavonoid intake and risk of esophageal squamous cell carcinoma: A population-based case-control study. Nutrition.

[19] Wang L.X., Shi Y.L., Zhang L.J., Wang K.R., Xiang L.P., Cai Z.Y., Lu J.L., Ye J.H., Liang Y.R., Zheng X.Q. (2019). Inhibitory Effects of (-)-Epigallocatechin-3-gallate on Esophageal Cancer. Molecules.

[20] Chacko S.M., Thambi P.T., Kuttan R., Nishigaki I. (2010). Beneficial effects of green tea: A literature review. Chin. Med..

[21] Atanasov A.G., Zotchev S.B., Dirsch V.M., Supuran C.T. (2021). Natural products in drug discovery: Advances and opportunities. Nat. Rev. Drug Discov..

[22] Jiang J.H., Pi J., Jin H., Yang F., Cai J.Y. (2018). Chinese herb medicine matrine induce apoptosis in human esophageal squamous cancer KYSE-150 cells through increasing reactive oxygen species and inhibiting mitochondrial function. Pathol. Res. Pract..

[23] Wang Q., Du H., Geng G., Zhou H., Xu M., Cao H., Zhang B., Song G., Hu T. (2014). Matrine inhibits proliferation and induces apoptosis via BID-mediated mitochondrial pathway in esophageal cancer cells. Mol. Biol. Rep..

[24] An K., Zhang Y., Liu Y., Yan S., Hou Z., Cao M., Liu G., Dong C., Gao J., Liu G. (2020). Neferine induces apoptosis by modulating the ROS-mediated JNK pathway in esophageal squamous cell carcinoma. Oncol. Rep..

[25] Zhu X., Li Z., Li T., Long F., Lv Y., Liu L., Liu X., Zhan Q. (2018). Osthole inhibits the PI3K/AKT signaling pathway via activation of PTEN and induces cell cycle arrest and apoptosis in esophageal squamous cell carcinoma. Biomed. Pharmacother..

[26] Yang J., Dou Z., Peng X., Wang H., Shen T., Liu J., Li G., Gao Y. (2019). Transcriptomics and proteomics analyses of anti-cancer mechanisms of TR35-An active fraction from Xinjiang Bactrian camel milk in esophageal carcinoma cell. Clin. Nutr..

[27] Almanaa T.N., Geusz M.E., Jamasbi R.J. (2012). Effects of curcumin on stem-like cells in human esophageal squamous carcinoma cell lines. BMC Complement. Altern. Med..

[28] Wang J.N., Che Y., Yuan Z.Y., Lu Z.L., Li Y., Zhang Z.R., Li N., Li R.D., Wan J., Sun H.D. (2019). Acetyl-macrocalin B suppresses tumor growth in esophageal squamous cell carcinoma and exhibits synergistic anti-cancer effects with the Chk1/2 inhibitor AZD7762. Toxicol. Appl. Pharmacol..

[29] Shi X.J., Ding L., Zhou W., Ji Y., Wang J., Wang H., Ma Y., Jiang G., Tang K., Ke Y. (2017). Pro-Apoptotic Effects of JDA-202, a Novel Natural Diterpenoid, on Esophageal Cancer Through Targeting Peroxiredoxin I. Antioxid. Redox Signal..

[30] Song M., Liu X., Liu K., Zhao R., Huang H., Shi Y., Zhang M., Zhou S., Xie H., Chen H. (2018). Targeting AKT with Oridonin Inhibits Growth of Esophageal Squamous Cell Carcinoma In Vitro and Patient-Derived Xenografts In Vivo. Mol. Cancer Ther..

[31] Ma Y.C., Ke Y., Zi X., Zhao W., Shi X.J., Liu H.M. (2013). Jaridonin, a novel ent-kaurene diterpenoid from Isodon rubescens, inducing apoptosis via production of reactive oxygen species in esophageal cancer cells. Curr. Cancer Drug Targets.

[32] Wang L., Lu A., Liu X., Sang M., Shan B., Meng F., Cao Q., Ji X. (2011). The flavonoid Baohuoside-I inhibits cell growth and downregulates survivin and cyclin D1 expression in esophageal carcinoma via β-catenin-dependent signaling. Oncol. Rep..

[33] Fan C., Yang Y., Liu Y., Jiang S., Di S., Hu W., Ma Z., Li T., Zhu Y., Xin Z. (2016). Icariin displays anticancer activity against human esophageal cancer cells via regulating endoplasmic reticulum stress-mediated apoptotic signaling. Sci. Rep..

[34] Ye L., Zhang J., Zhang Y., Gu B., Zhu H., Mao X. (2020). Isoliquiritigenin Suppressed Esophageal Squamous Carcinoma Growth by Blocking EGFR Activation and Inducing Cell Cycle Arrest. BioMed Res. Int..

[35] Guo F., Hu Y., Niu Q., Li Y., Ding Y., Ma R., Wang X., Li S., Xie J. (2018). Grape Seed Proanthocyanidin Extract Inhibits Human Esophageal Squamous Cancerous Cell Line ECA109 via the NF-κB Signaling Pathway. Mediat. Inflamm..

[36] Zheng N.G., Mo S.J., Li J.P., Wu J.L. (2014). Anti-CSC effects in human esophageal squamous cell carcinomas and Eca109/9706 cells induced by nanoliposomal quercetin alone or combined with CD 133 antiserum. Asian Pac. J. Cancer Prev..

[37] Gao J., Xia R., Chen J., Gao J., Luo X., Ke C., Ren C., Li J., Mi Y. (2020). Inhibition of esophageal-carcinoma cell proliferation by genistein via suppression of JAK1/2-STAT3 and AKT/MDM2/p53 signaling pathways. Aging.

[38] Huang P., Sun L.Y., Zhang Y.Q. (2019). A Hopeful Natural Product, Pristimerin, Induces Apoptosis, Cell Cycle Arrest, and Autophagy in Esophageal Cancer Cells. Anal. Cell. Pathol..

[39] Wu Y., Zhu Y., Li S., Zeng M., Chu J., Hu P., Li J., Guo Q., Lv X.B., Huang G. (2017). Terrein performs antitumor functions on esophageal cancer cells by inhibiting cell proliferation and synergistic interaction with cisplatin. Oncol. Lett..

[40] Lu Z., Zhang G., Zhang Y., Hua P., Fang M., Wu M., Liu T. (2018). Isoalantolactone induces apoptosis through reactive oxygen species-dependent upregulation of death receptor 5 in human esophageal cancer cells. Toxicol. Appl. Pharmacol..

[41] Zhang R., Hao J., Guo K., Liu W., Yao F., Wu Q., Liu C., Wang Q., Yang X. (2020). Germacrone Inhibits Cell Proliferation and Induces Apoptosis in Human Esophageal Squamous Cell Carcinoma Cells. BioMed Res. Int..

[42] Meng X., Dong X., Wang W., Yang L., Zhang X., Li Y., Chen T., Ma H., Qi D., Su J. (2018). Natural Borneol Enhances Paclitaxel-Induced Apoptosis of ESCC Cells by Inactivation of the PI3K/AKT. J. Food Sci..

[43] Ma J., Hu X., Liao C., Xiao H., Zhu Q., Li Y., Liu Z., Tao A., He Z., Xu C. (2019). Gypenoside L Inhibits Proliferation of Liver and Esophageal Cancer Cells by Inducing Senescence. Molecules.

[44] Yan S., Tian S., Kang Q., Xia Y., Li C., Chen Q., Zhang S., Li Z. (2015). Rhizoma Paridis Saponins Suppresses Tumor Growth in a Rat Model of N-Nitrosomethylbenzylamine-Induced Esophageal Cancer by Inhibiting Cyclooxygenases-2 Pathway. PLoS ONE.

[45] Vahedi Larijani L., Ghasemi M., AbedianKenari S., Naghshvar F. (2014). Evaluating the effect of four extracts of avocado fruit on esophageal squamous carcinoma and colon adenocarcinoma cell lines in comparison with peripheral blood mononuclear cells. Acta Med. Iran..

[46] Wang L.S., Dombkowski A.A., Seguin C., Rocha C., Cukovic D., Mukundan A., Henry C., Stoner G.D. (2011). Mechanistic basis for the chemopreventive effects of black raspberries at a late stage of rat esophageal carcinogenesis. Mol. Carcinog..

[47] Kresty L.A., Weh K.M., Zeyzus-Johns B., Perez L.N., Howell A.B. (2015). Cranberry proanthocyanidins inhibit esophageal adenocarcinoma in vitro and in vivo through pleiotropic cell death induction and PI3K/AKT/mTOR inactivation. Oncotarget.

[48] Zhao L., Yan X., Shi J., Ren F., Liu L., Sun S., Shan B. (2015). Ethanol extract of Forsythia suspensa root induces apoptosis of esophageal carcinoma cells via the mitochondrial apoptotic pathway. Mol. Med. Rep..

[49] Li C., Zhang Z., Zhang S., Yan W., Si C., Lee M.H., Li Z. (2019). Inhibitory Effects of the Extracts of Juglans sigillata Green Husks on the Proliferation, Migration and Survival of KYSE150 and EC9706 Human Esophageal Cancer Cell Lines. Nutr. Cancer.

[50] Fan W., Sun L., Zhou J.Q., Zhang C., Qin S., Tang Y., Liu Y., Lin S.S., Yuan S.T. (2015). Marsdenia tenacissima extract induces G0/G1 cell cycle arrest in human esophageal carcinoma cells by inhibiting mitogen-activated protein kinase (MAPK) signaling pathway. Chin. J. Nat. Med..

[51] Li F.R., Jiao P., Yao S.T., Sang H., Qin S.C., Zhang W., Zhang Y.B., Gao L.L. (2012). Paris polyphylla Smith extract induces apoptosis and activates cancer suppressor gene connexin26 expression. Asian Pac. J. Cancer Prev..

[52] Peiffer D.S., Zimmerman N.P., Wang L.S., Ransom B.W., Carmella S.G., Kuo C.T., Siddiqui J., Chen J.H., Oshima K., Huang Y.W. (2014). Chemoprevention of esophageal cancer with black raspberries, their component anthocyanins, and a major anthocyanin metabolite, protocatechuic acid. Cancer Prev. Res..

[53] Hadisaputri Y.E., Miyazaki T., Suzuki S., Kubo N., Zuhrotun A., Yokobori T., Abdulah R., Yazawa S., Kuwano H. (2015). Molecular characterization of antitumor effects of the rhizome extract from Curcuma zedoaria on human esophageal carcinoma cells. Int. J. Oncol..

[54] Skerman N.B., Joubert A.M., Cronjé M.J. (2011). The apoptosis inducing effects of Sutherlandia spp. extracts on an oesophageal cancer cell line. J. Ethnopharmacol..

[55] Nagata T., Toume K., Long L.X., Hirano K., Watanabe T., Sekine S., Okumura T., Komatsu K., Tsukada K. (2016). Anticancer effect of a Kampo preparation Daikenchuto. J. Nat. Med..

[56] Liu W., Li S.Y., Huang X.E., Cui J.J., Zhao T., Zhang H. (2012). Inhibition of tumor growth in vitro by a combination of extracts from Rosa roxburghii Tratt and Fagopyrum cymosum. Asian Pac. J. Cancer Prev..

[57] Jia Y.S., Hu X.Q., Li J.A., Andras S., Hegyi G., Han B.S. (2016). Tonglian Decoction () arrests the cell cycle in S-phase by targeting the nuclear factor-kappa B signal pathway in esophageal carcinoma Eca109 cells. Chin. J. Integr. Med..

[58] Chen W.K., Chen C.A., Chi C.W., Li L.H., Lin C.P., Shieh H.R., Hsu M.L., Ko C.C., Hwang J.J., Chen Y.J. (2019). Moscatilin Inhibits Growth of Human Esophageal Cancer Xenograft and Sensitizes Cancer Cells to Radiotherapy. J. Clin. Med..

[59] Medda R., Lyros O., Schmidt J.L., Jovanovic N., Nie L., Link B.J., Otterson M.F., Stoner G.D., Shaker R., Rafiee P. (2015). Anti inflammatory and anti angiogenic effect of black raspberry extract on human esophageal and intestinal microvascular endothelial cells. Microvasc. Res..

[60] Huang Y.W., Gu F., Dombkowski A., Wang L.S., Stoner G.D. (2016). Black raspberries demethylate Sfrp4, a WNT pathway antagonist, in rat esophageal squamous cell papilloma. Mol. Carcinog..

[61] Shi Q., Diao Y., Jin F., Ding Z. (2018). Anti-metastatic effects of Aidi on human esophageal squamous cell carcinoma by inhibiting epithelial-mesenchymal transition and angiogenesis. Mol. Med. Rep..

[62] Xu W.W., Zheng C.C., Huang Y.N., Chen W.Y., Yang Q.S., Ren J.Y., Wang Y.M., He Q.Y., Liao H.X., Li B. (2018). Synephrine Hydrochloride Suppresses Esophageal Cancer Tumor Growth and Metastatic Potential through Inhibition of Galectin-3-AKT/ERK Signaling. J. Agric. Food Chem..

[63] Wang J., Wu M., Zheng D., Zhang H., Lv Y., Zhang L., Tan H.S., Zhou H., Lao Y.Z., Xu H.X. (2020). Garcinol inhibits esophageal cancer metastasis by suppressing the p300 and TGF-β1 signaling pathways. Acta Pharmacol. Sin..

[64] Gu Z.F., Zhang Z.T., Wang J.Y., Xu B.B. (2017). Icariin exerts inhibitory effects on the growth and metastasis of KYSE70 human esophageal carcinoma cells via PI3K/AKT and STAT3 pathways. Environ. Toxicol. Pharmacol..

[65] Li H., Yao Q., Min L., Huang S., Wu H., Yang H., Fan L., Wang J., Zheng N. (2020). The Combination of Two Bioactive Constituents, Lactoferrin and Linolenic Acid, Inhibits Mouse Xenograft Esophageal Tumor Growth by Downregulating Lithocholyltaurine and Inhibiting the JAK2/STAT3-Related Pathway. ACS Omega.

[66] Yan H., Wang X., Wang Y., Wang P., Xiao Y. (2014). Antiproliferation and anti-migration induced by gypenosides in human colon cancer SW620 and esophageal cancer Eca-109 cells. Hum. Exp. Toxicol..

[67] Ding Z., Lao Y., Zhang H., Fu W., Zhu L., Tan H., Xu H. (2016). Griffipavixanthone, a dimeric xanthone extracted from edible plants, inhibits tumor metastasis and proliferation via downregulation of the RAF pathway in esophageal cancer. Oncotarget.

[68] Batirel S., Yilmaz A.M., Sahin A., Perakakis N., Kartal Ozer N., Mantzoros C.S. (2018). Antitumor and antimetastatic effects of walnut oil in esophageal adenocarcinoma cells. Clin. Nutr..

[69] Liu Y.M., Liu Y.K., Huang P.I., Tsai T.H., Chen Y.J. (2018). Antrodia cinnamomea mycelial fermentation broth inhibits the epithelial-mesenchymal transition of human esophageal adenocarcinoma cancer cells. Food Chem. Toxicol..

[70] Li L., Yue G.G., Lee J.K., Wong E.C., Fung K.P., Yu J., Lau C.B., Chiu P.W. (2017). The adjuvant value of Andrographis paniculata in metastatic esophageal cancer treatment—From preclinical perspectives. Sci. Rep..

[71] Xie X., Zu X., Liu F., Wang T., Wang X., Chen H., Liu K., Wang P., Liu F., Zheng Y. (2019). Purpurogallin is a novel mitogen-activated protein kinase kinase 1/2 inhibitor that suppresses esophageal squamous cell carcinoma growth in vitro and in vivo. Mol. Carcinog..

[72] Shi H., Shi D., Wu Y., Shen Q., Li J. (2016). Qigesan inhibits migration and invasion of esophageal cancer cells via inducing connexin expression and enhancing gap junction function. Cancer Lett..

[73] Kong L., Wu Z., Zhao Y., Lu X., Shi H., Liu S., Li J. (2019). Qigesan reduces the motility of esophageal cancer cells via inhibiting Gas6/Axl and NF-κB expression. Biosci. Rep..

[74] Wang T.H., Wan J.Y., Gong X., Li H.Z., Cheng Y. (2012). Tetrandrine enhances cytotoxicity of cisplatin in human drug-resistant esophageal squamous carcinoma cells by inhibition of multidrug resistance-associated protein 1. Oncol. Rep..

[75] Lightdale C. (2005). Defined Green Tea Catechin Extract in Preventing Esophageal Cancer in Patients with Barrett’s Esophagus. https://ClinicalTrials.gov/show/NCT00233935.

[76] Shan G.Y., Zhang S., Li G.W., Chen Y.S., Liu X.A., Wang J.K. (2011). Clinical evaluation of oral Fructus bruceae oil combined with radiotherapy for the treatment of esophageal cancer. Chin. J. Integr. Med..

[77] American Cancer Society (2020). If You Have Esophagus Cancer. https://www.cancer.org/cancer/esophagus-cancer/if-you-have-esophagus-cancer.html.

[78] Housman G., Byler S., Heerboth S., Lapinska K., Longacre M., Snyder N., Sarkar S. (2014). Drug resistance in cancer: An overview. Cancers.

[79] Mansoori B., Mohammadi A., Davudian S., Shirjang S., Baradaran B. (2017). The Different Mechanisms of Cancer Drug Resistance: A Brief Review. Adv. Pharm. Bull..

[80] Altun İ., Sonkaya A. (2018). The Most Common Side Effects Experienced by Patients Were Receiving First Cycle of Chemotherapy. Iran. J. Public Health.

[81] World Cancer Research Fund International (2020). Oesophageal Cancer Statistics. https://www.wcrf.org/cancer-trends/oesophageal-cancer-statistics.

